# Epidemiological Decoupling of COVID-19 in Pernambuco, Brazil (2020–2023): A Subnational Time-Series Risk Analysis Using Generalized Models

**DOI:** 10.3390/epidemiologia7050134

**Published:** 2026-09-26

**Authors:** Yasmin Esther Barreto, Matheus Paiva Emidio Cavalcanti, Rosalina Semedo de Andrade Tavares, Alexandre Castelo Branco Araujo, Andréa Vasconcellos Batista da Silva, Fernando Augusto Marinho Dos Santos Figueira, Eric Shamus, Weverton Pereira de Medeiros, Carlos Mendes Tavares, Luiz Carlos de Abreu

**Affiliations:** 1 Department of Public Health, University of Limerick, V94 T9PX Limerick, Ireland; cdh.fsp@gmail.com; 2 Postgraduate Program in Medical Sciences, Faculty of Medicine, University of São Paulo, São Paulo 01246-903, SP, Brazil; alexandrecbranco@terra.com.br (A.C.B.A.); avasconcellosbatista@usp.br (A.V.B.d.S.); fernando@imip.org.br (F.A.M.D.S.F.); 3 Laboratory of Design of Studies and Scientific Writing, Federal University of Espírito Santo, Vitória 29075-910, ES, Brazil; mpaivaemidio@gmail.com (M.P.E.C.); wevertonmedeiros74@gmail.com (W.P.d.M.); 4 Institute of Applied Social Sciences, University of International Integration of Afro-Brazilian Lusophony (UNILAB), Redenção 62790-000, CE, Brazil; rosalina@unilab.edu.br (R.S.d.A.T.); carlostavares@unilab.edu.br (C.M.T.); 5 Rehabilitation Sciences, Florida Gulf Coast University (FGCU), 10501 FGCU Blvd. S., Fort Myers, FL 33965, USA; eshamus@fgcu.edu; 6 Graduate Program in Public Health, Federal University of Espírito Santo, Vitória 29075-910, ES, Brazil

**Keywords:** COVID-19, epidemiology, time series studies, incidence, mortality, case fatality rate, Generalized Linear Models, Generalized Additive Models, health surveillance, Pernambuco, Brazil

## Abstract

**Background/Objectives:** COVID-19 placed substantial pressure on health systems and showed marked spatial and temporal heterogeneity in Brazil. We analyzed monthly and annual patterns of incidence, mortality, and period-specific case fatality in Pernambuco from 2020 to 2023. **Methods:** This ecological time-series study used 48 monthly surveillance observations. Monthly case and death counts were modeled with calendar year as a categorical predictor. Negative binomial generalized linear models with log offsets were selected as the primary models because Poisson models showed marked overdispersion; heteroscedasticity—and autocorrelation-consistent (HAC) standard errors were used to reduce sensitivity to serial dependence. A log-linear model estimated Monthly Percent Change (MPC) using a four-month moving-block bootstrap. A quasi-Poisson generalized additive model (GAM) with k = 10 was examined as an exploratory description of non-linearity. **Results:** Annual incidence increased from 2310.85 per 100,000 in 2020 to 4905.65 per 100,000 in 2022 and decreased to 908.18 per 100,000 in 2023. Mortality peaked in 2021 and period-specific CFR declined markedly thereafter. In the primary negative binomial models with HAC inference, incidence was lower in 2023 than in 2020 (RR = 0.39; 95% CI: 0.17–0.89; *p* = 0.0247). Mortality in 2021 did not differ from 2020 (RR = 1.11; 95% CI: 0.43–2.89; *p* = 0.8270), whereas mortality was lower in 2022 (RR = 0.22; 95% CI: 0.09–0.52; *p* = 0.0006) and 2023 (RR = 0.06; 95% CI: 0.03–0.14; *p* < 0.0001). The global MPC was +2.52% (moving-block bootstrap 95% CI: −5.42% to 11.00%; *p* = 0.5152), providing no evidence of a consistent global log-linear trend. The exploratory GAM suggested a non-linear trajectory, but its basis-dimension check indicated that k = 10 was insufficient (k-index = 0.469; *p* < 0.001); therefore, no inferential conclusion was based on the GAM. **Conclusions:** Pernambuco surveillance data show an epidemiological divergence between reported incidence and severe outcomes, particularly from 2022 onward. Because vaccination, hybrid immunity, circulating variants, testing practices, and clinical management were not directly modeled, these findings support epidemiological decoupling but do not by themselves establish its biological mechanisms.

## 1. Introduction

The emergence of Severe Acute Respiratory Syndrome Coronavirus 2 (SARS-CoV-2) in late 2019 triggered one of the most severe public health emergencies of the 21st century [1]. In January 2020, the World Health Organization (WHO) issued its first Situation Report on COVID-19, highlighting the alarming rise in cases in Wuhan, China, and other countries. This led to a risk re-evaluation and the formal classification of the event as a Public Health Emergency of International Concern (PHEIC). That same month, 18 countries had already confirmed cases of the disease, prompting the Brazilian Ministry of Health to declare a Public Health Emergency of National Importance (ESPIN) on February 3 due to the rapid spread of the disease [2,3,4].

Brazil spans a vast territory divided into 26 states and the Federal District, organized into five distinct macro-regions. Following confirmation of the initial cases, intense community transmission swept through all regions [5]. Marked socioeconomic, demographic, and healthcare infrastructure disparities among states resulted in deeply uneven spatiotemporal transmission patterns and disease burdens [6]. This geographic and structural heterogeneity directly influenced public adherence to preventive strategies throughout the pandemic, emphasizing the need for subnational analyses to capture regional epidemiological specificities [7].

The state of Pernambuco, in Northeastern Brazil, was among the most severely impacted subnational units in the early pandemic phases, exhibiting high incidence and mortality rates [8]. With a population density of 92.37 inhabitants/km^2^ (2022 IBGE Census), the state has undergone notable demographic shifts, including population aging and a declining fertility rate; individuals aged ≥ 60 years now comprise over 15% of the population. In urban centers, particularly the Recife Metropolitan Area, these demographic vulnerabilities interacted with high population density, intense tourist flow, socioeconomic disparities, and unequal healthcare access. Coupled with the premature relaxation of containment measures prior to the January 2021 vaccination rollout, these structural and demographic factors significantly exacerbated local transmission dynamics across successive viral waves [9].

In addition to the heterogeneous dissemination of COVID-19, viral diversification occurred unevenly across settings. Movement restrictions, border controls, mitigation measures, vaccination, and access to testing and care varied over time and space [10]. The Omicron lineage was notable for high transmissibility: BA.1 drove a major wave from late 2021 into early 2022, and BA.2 subsequently became predominant during the first quarter of 2022 [11,12].

Because they combine high transmissibility with a preferential tropism for the upper respiratory tract (rather than the lower respiratory tract), the absolute number of diagnoses rose exponentially without being proportionally accompanied by hospitalizations and deaths. Conceição et al. (2025) [13] longitudinally demonstrated the decoupling of COVID-19 in Brazil, where, following the implementation of pharmacological strategies alongside Omicron sublineages, incidence reached historical highs while hospitalization and mortality rates fell by more than 90%, highlighting the loss of synchrony between these indicators. Other studies describe the pandemic’s trajectories and the influence of waves, noting a decline in severity and hypothesizing a temporal relationship with vaccination interventions [14,15]. The mechanisms underlying that divergence cannot be identified from aggregated surveillance indicators alone.

Evaluating the temporal trajectory of COVID-19 requires standardized epidemiological metrics capable of monitoring different dimensions of disease burden. Incidence Rates (IRs), Mortality Rates (MRs), and Case Fatality Rates (CFRs) serve as fundamental indicators to evaluate simultaneously transmission intensity, population-level mortality burden, and clinical severity among confirmed cases [16,17].

Beyond descriptive epidemiology, inferential statistical methods are essential to quantify relative risk variations across different temporal phases. Generalized Linear Models (GLMs) are widely used in epidemiological research due to their flexibility in modeling count data and estimating rate ratios adjusted for population offsets [18]. Complementarily, time-series regression approaches, including log-linear models for Monthly Percent Change (MPC) estimation and Generalized Additive Models (GAMs) for non-linear trend identification, provide robust frameworks to unravel complex epidemic oscillations and wave structures [19].

Although national-level studies have documented COVID-19 in Brazil, detailed state-level time-series analyses remain important for describing regional trajectories [20]. This study therefore analyzed monthly temporal patterns and between-year differences in reported COVID-19 incidence, mortality, and period-specific case fatality in Pernambuco from 2020 to 2023. We hypothesized that the surveillance series would show an epidemiological divergence between reported incidence and severe outcomes after the initial waves, while recognizing that the ecological design could not directly attribute such divergence to vaccination, variants, immunity, or changes in care.

## 2. Materials and Methods

### 2.1. Study Design and Data Sources

This ecological time-series study analyzed aggregated secondary data on COVID-19 in the state of Pernambuco, Brazil, spanning from 1 January 2020, to 31 December 2023. The ecological design allowed the evaluation of population-level indicators and captured long-term epidemiological risk transitions across distinct pandemic phases.

The study extracted official secondary surveillance data from consolidated public health information systems managed by the Brazilian Ministry of Health and the State Health Secretariat of Pernambuco. Annual population estimates from the Brazilian Institute of Geography and Statistics (IBGE), derived from official projection models, served as denominators for rate calculations. Finally, the analytical pipeline structured the data at both monthly and annual resolutions to support descriptive, trend, and inferential modeling.

### 2.2. Unit of Analysis and Temporal Aggregation

The observational unit for all inferential models was the calendar month (*N* = 48 consecutive monthly observations). Confirmed case and death counts were aggregated for each month from January 2020 through December 2023. Calendar year (2020, 2021, 2022, or 2023) was then assigned to each monthly observation and entered as a categorical predictor, with 2020 as the reference. Annual totals and rates (*N* = 4 calendar-year summaries) were used only for descriptive presentation and were not treated as four independent observations in the GLMs. The same monthly series was used for descriptive distributions, log-linear trend analysis, and GAM smoothing.

### 2.3. Epidemiological Indicators and Mathematical Definitions

Three core-standardized indicators were constructed:

$$Incidence\;Rate\;\left(IR\right):\;\mathrm{IR}=\left(\frac{\mathrm{New}\;\mathrm{Confirmed}\;\mathrm{Cases}\;\mathrm{in}\;\mathrm{Period}}{\mathrm{Resident}\;\mathrm{Population}\;\mathrm{in}\;\mathrm{Period}}\right)\times 100,000$$

$$Mortality\;Rate\;\left(MR\right):\;\mathrm{MR}=\left(\frac{\mathrm{Confirmed}\;\mathrm{Deaths}\;\mathrm{in}\;\mathrm{Period}}{\mathrm{Resident}\;\mathrm{Population}\;\mathrm{in}\;\mathrm{Period}}\right)\times 100,000$$

$$Case\;Fatality\;Rate\;\left(CFR\right):\;\mathrm{CFR}=\left(\frac{\mathrm{Confirmed}\;\mathrm{Deaths}\;\mathrm{in}\;\mathrm{Period}}{\mathrm{New}\;\mathrm{Confirmed}\;\mathrm{Cases}\;\mathrm{in}\;\mathrm{Period}}\right)\times 100$$

For period-specific CFR modeling, deaths in a given calendar month were related to confirmed cases reported in the same month. A continuity correction of 0.5 was added to the case-count offset to include January and February 2020, the only two months with zero reported cases (and zero reported deaths). A sensitivity analysis that excluded these two months and used log(cases) as the offset produced virtually unchanged negative binomial estimates (e.g., 2021 versus 2020: RR = 0.558 with the correction and RR = 0.558 after exclusion). This period-specific indicator does not link deaths to a defined cohort of cases and may be affected by the delay between case confirmation and death.

### 2.4. Descriptive Statistics

Descriptive statistics of monthly observations were computed to summarize central tendency and dispersion. Given the marked skewness and overdispersion inherent to epidemic time series with recurrent wave peaks, medians and Interquartile Ranges (IQR, 25th–75th percentiles) were reported.

### 2.5. Inferential Risk Modeling (Generalized Linear Models—GLM)

Between-year rate ratios were estimated from monthly count data using generalized linear count models. Monthly cases and deaths were the dependent variables for incidence and mortality, respectively, with log(monthly resident population) as the offset. For period-specific case fatality, monthly deaths were modeled with log(monthly confirmed cases + 0.5) as the offset. Calendar year was included as a categorical predictor, with 2020 as the reference category. Thus, the models contained 48 monthly observational units rather than four annual observations.

Poisson models with a log link were fitted first and compared with negative binomial models. Overdispersion was evaluated using Pearson dispersion and residual deviance relative to residual degrees of freedom, and model fit was compared using Akaike’s Information Criterion (AIC). Poisson dispersion was extreme for incidence (Pearson dispersion = 15,871.5), mortality (327.7), and period-specific case fatality (103.7). Negative binomial models substantially improved AIC for incidence (1059.0 versus 702,393.5), mortality (648.7 versus 15,530.6), and case fatality (577.2 versus 4620.9); they were therefore designated as the primary models. Pearson residual diagnostics still indicated temporal autocorrelation: the lag-12 Ljung–Box test yielded *p* = 0.0039 for incidence, *p* < 0.0001 for mortality, and *p* < 0.0001 for period-specific CFR. Inference therefore used HAC standard errors with three monthly lags. Conventional Poisson and negative binomial estimates are retained as sensitivity analyses in the Supplementary Materials. Results are reported as rate ratios (RRs), 95% confidence intervals (CIs), and two-sided *p*-values.

### 2.6. Temporal Trend Analysis (Log-Linear and GAM)

The overall temporal trend of monthly incidence was evaluated via log-linear regression:

$$\ln\left(Y_{t}\right)=\alpha +\beta \cdot t$$

where $Y_{t}$ represents the incidence rate at month t (t=1,2,…,48) and β is the slope coefficient. The Monthly Percent Change (MPC) was derived as:

$$\mathrm{MPC}=\left[\exp\left(\beta \right)-1\right]\times 100$$

Because the first two months had zero reported cases, the log-linear analysis used log[(monthly cases + 0.5)/population × 100,000]. Residual temporal dependence was examined using autocorrelation and partial-autocorrelation functions and the Ljung–Box test. Uncertainty around the MPC was estimated with 10,000 circular moving-block bootstrap replicates, a fixed random seed, and a block length of four months (approximately *N*^(1/3)), thereby resampling short consecutive segments rather than treating months as exchangeable. A sensitivity analysis excluded the two zero-case months and fitted the uncorrected log rate.

To explore non-linear oscillations, a generalized additive model (GAM) was fitted to monthly case counts using a quasi-Poisson family, log link, log (population) offset, restricted maximum likelihood (REML), and a penalized thin-plate regression spline for time. The basis dimension k = 10 was initially selected to allow multiple epidemic waves across 48 months while limiting maximum complexity. The fitted smooth had 3.891 effective degrees of freedom and explained 47.8% of deviance. However, the subsequent basis-dimension diagnostic was unfavorable (k′ = 9; k-index = 0.469; *p* < 0.001), indicating residual pattern and suggesting that k = 10 may have been insufficient. Accordingly, the GAM is retained only as an exploratory visualization; its smooth-term significance and precise shape are not used for inferential conclusions or causal attribution.

$$g\left(E\left(Y_{t}\right)\right)=\alpha +s\left(t\right)$$

where s(t) is the penalized smooth function of month and g(·) is the log link. Observed and fitted counts were converted to rates per 100,000 inhabitants for graphical presentation.

### 2.7. Computational Environment and Ethics

All processing, data cleaning, statistical modeling, and graphical rendering were executed in the R statistical environment on the R version 4.6.1 (R Foundation for Statistical Computing, Vienna, Austria) [21].

Ethical Statement: This study relied exclusively on secondary, aggregated, fully anonymized public domain data. In accordance with Brazilian National Health Council (CNS) Resolutions No. 466/2012, 510/2016, and 738/2024, formal ethics committee approval was waived.

## 3. Results

### 3.1. Descriptive Dynamics, Annual Aggregation, and Temporal Trajectories

Between January 2020 and December 2023, Pernambuco recorded 1,211,807 confirmed COVID-19 cases and 23,192 deaths. Annual aggregated indicators demonstrated contrasting temporal trajectories between transmission and severity over time (Table 1).

The 48 monthly observations showed substantial dispersion associated with recurrent epidemic waves (Table 2, Figure 1). Median monthly incidence was 183.73 per 100,000 (IQR: 76.55–397.26), median monthly mortality was 1.98 per 100,000 (IQR: 0.68–6.48), and median monthly period-specific CFR was 1.62% (IQR: 0.52–2.75%). The surveillance trajectories show that high reported incidence in early 2022 coincided with substantially lower mortality and case fatality than in the initial waves. This descriptive divergence is referred to here as epidemiological decoupling; it does not directly demonstrate a biological mechanism.

Values are reported as medians and interquartile ranges (IQRs) and do not assume a probability distribution. Inferential analyses used the 48 monthly observations. Primary models were negative binomial GLMs with HAC standard errors: cases with log(population) offset for incidence, deaths with log(population) offset for mortality, and deaths with log(cases + 0.5) offset for period-specific case fatality. Poisson estimates are reported only as sensitivity analyses because their standard errors were severely underestimated under overdispersion.

The three indicators are presented in separate panels because incidence and mortality are expressed as rates per 100,000 inhabitants, whereas case fatality is expressed as a percentage. The panels show monthly values from January 2020 through December 2023.

### 3.2. Annual Risk Comparisons (Generalized Linear Models)

Negative binomial GLMs were fitted to the 48 monthly observations, with 2020 as the reference year and HAC standard errors to account for residual serial dependence, to quantify between-year differences in the three surveillance indicators (Table 3). Point estimates were similar to the corresponding annual rate ratios, but uncertainty was substantially wider than under Poisson models.

For reported incidence, the estimated rate ratios for 2021 (RR = 1.90; 95% CI: 0.82–4.38; *p* = 0.1327) and 2022 (RR = 2.13; 95% CI: 0.93–4.86; *p* = 0.0736) did not provide evidence of a difference from 2020. Reported incidence was lower in 2023 (RR = 0.39; 95% CI: 0.17–0.89; *p* = 0.0247). These inferences use the primary negative binomial model and HAC standard errors; the much narrower Poisson intervals were not used because of severe overdispersion.

Mortality in 2021 did not differ statistically from 2020 in the primary model (RR = 1.11; 95% CI: 0.43–2.89; *p* = 0.8270). Mortality was lower in 2022 (RR = 0.22; 95% CI: 0.09–0.52; *p* = 0.0006) and 2023 (RR = 0.06; 95% CI: 0.03–0.14; *p* < 0.0001). Period-specific case fatality was also lower than in 2020 in 2021 (RR = 0.56; 95% CI: 0.32–0.96; *p* = 0.0363), 2022 (RR = 0.13; 95% CI: 0.07–0.25; *p* < 0.0001), and 2023 (RR = 0.21; 95% CI: 0.10–0.47; *p* = 0.0001). The continuity-correction sensitivity analysis produced nearly identical CFR estimates (Figure 2).

### 3.3. Temporal Trends and Non-Linear Modeling

The global log-linear analysis estimated an MPC of +2.52% (four-month moving-block bootstrap 95% CI: −5.42% to 11.00%; *p* = 0.5152) (Table 4). Because the interval included zero, these data provide no evidence of a consistent global log-linear trend across the full 48-month series. This should not be interpreted as proof that incidence was stable: the observed trajectory was strongly oscillatory, and residual autocorrelation was substantial. In a sensitivity analysis excluding January and February 2020, which had zero reported cases, the MPC was −2.97% (conventional 95% CI: −5.72% to −0.14%; *p* = 0.0402), showing that a single global slope is was sensitive to the treatment of the pre-epidemic zero months.

The exploratory GAM smooth suggested non-linear variation, with higher fitted incidence across 2021–2022 and lower fitted values through much of 2023 (Figure 3). The model’s smooth term had 3.891 effective degrees of freedom, and the model explained 47.8% of deviance. Nevertheless, the k-check was unfavorable (k-index = 0.469; *p* < 0.001), indicating that k = 10 did not adequately rule out remaining temporal pattern. Figure 3 is therefore presented only as an exploratory visualization. Neither the apparent peak locations nor the smooth-term *p*-value is used as confirmatory evidence.

Monthly case counts were modeled using a GAM with a quasi-Poisson family, log link, log(population) offset, REML estimation, and a penalized thin-plate regression spline [s(time, k = 10)]. Model-predicted counts were converted to incidence rates per 100,000 inhabitants. Points represent observed monthly incidence and the solid line represents the fitted exploratory smooth. The basis-dimension diagnostic indicated potential underfitting (k-index = 0.469; *p* < 0.001); consequently, the curve is descriptive and should not be used to identify exact inflection points, variant-specific waves, or causal mechanisms.

## 4. Discussion

Between January 2020 and December 2023, Pernambuco recorded 1,211,807 confirmed cases and 23,192 COVID-19 deaths. The principal finding is an epidemiological divergence in the aggregated surveillance series: reported incidence reached its annual peak in 2022, while mortality and period-specific case fatality were already substantially lower than in 2020 (Figure 1). In the primary negative binomial models with HAC inference, mortality in 2021 was not statistically distinguishable from 2020, whereas mortality and period-specific case fatality were lower from 2022 onward. This pattern supports epidemiological decoupling in the observed indicators but cannot, by itself, establish biological decoupling or identify its mechanisms.

This temporal dissociation should be interpreted within the evolving epidemiological context of COVID-19. Early evidence documented the rapid dissemination of SARS-CoV-2 and its implications for global health [22,23]. Subsequent comparative evidence showed a lower risk of hospitalization and death associated with Omicron than with Delta [14], while genomic and epidemiological data documented changes in the circulation of SARS-CoV-2 variants in Northeastern Brazil [11]. However, because vaccination coverage, previous infection, and circulating variants were not directly incorporated into the present models, their individual contributions to the epidemiological decoupling observed in Pernambuco cannot be determined [24] (Figure 1).

Pernambuco’s early pandemic trajectory occurred within the broader context of social vulnerability and health-system pressure documented in Northeastern Brazil [10,11,15]. In the present study, mortality rates reached 100.42 deaths per 100,000 inhabitants in 2020 and 111.60 per 100,000 in 2021. Evidence from another Northeastern Brazilian setting also showed differences in post-vaccination seroprevalence and infection between municipalities with higher and lower population-level vaccination coverage [25]. Although these findings provide relevant regional context, they do not directly explain the temporal patterns observed in Pernambuco.

Despite broad alignment with global trends, key temporal features of Pernambuco’s trajectory diverge from patterns reported in high-income nations and other Brazilian regions [6]. In many North American and European settings, peak incidence occurred during earlier variant surges, followed by a contraction in transmission driven by early public health interventions, robust healthcare responses, and rapid vaccine deployment among populations with greater healthcare access and socioeconomic capital [13,14].

In contrast, Pernambuco recorded its highest annual reported incidence in 2022 (Figure 2). This descriptive peak coincided temporally with the circulation of Omicron subvariants, particularly BA.1 and BA.2 [12]. However, variant-specific data were not incorporated into the models, precluding direct attribution of the observed pattern to these lineages [26].

Pernambuco exhibited a marked overall reduction in period-specific case fatality across the study period, declining from 4.35% in 2020 to 0.45% in 2022, followed by a modest increase to 0.69% in 2023. Despite this increase, the 2023 estimate remained substantially lower than those observed during the initial pandemic years. Changes in testing availability and eligibility across pandemic phases may have influenced these estimates. However, testing practices were not directly evaluated in the present study, and this explanation remains hypothetical.

Several mechanisms could plausibly contribute to the observed epidemiological divergence, including vaccination, hybrid immunity, changes in circulating variants, improved clinical management, and changes in the age and risk profile of detected cases. These factors were not directly included in the present models. Accordingly, the 2022 findings—lower mortality (RR = 0.22; 95% CI: 0.09–0.52) and lower period-specific case fatality (RR = 0.13; 95% CI: 0.07–0.25), despite the highest annual reported incidence—should be interpreted as population-level epidemiological decoupling rather than evidence of a specific biological pathway.

Nyberg et al. (2022) [12] reported lower risks of hospitalization and death associated with the Omicron variant compared with the Delta variant in England. Although these findings provide relevant context for interpreting the reduction in severe outcomes during the Omicron period, differences in population immunity, vaccination coverage, healthcare access, and surveillance practices limit their direct extrapolation to Pernambuco. Similar epidemiological findings were documented in Northeastern Brazil by Cavalcanti et al. (2026) [27], who observed a parallel dissociation between reported transmission and severe outcomes in Ceará during 2022, coinciding with the circulation of Omicron BA.1 and BA.2. Given the methodological limitations of ecological data for establishing variant-specific causality, further studies integrating epidemiological indicators, vaccination coverage, hospitalization data, and diagnostically confirmed variant profiles are warranted.

The lower reported incidence in 2023 (RR = 0.39; 95% CI: 0.17–0.89) also requires caution. After the WHO ended the Public Health Emergency of International Concern in May 2023 and surveillance practices evolved, reduced testing of mild ambulatory cases may have contributed to fewer notifications. Mortality was also lower in 2023 (RR = 0.06; 95% CI: 0.03–0.14), but mortality data are not entirely immune to changes in ascertainment, certification, and reporting. The combined indicators are consistent with a lower burden of severe reported disease, but they do not independently validate a biological mechanism.

Methodologically, the results show why model choice matters in epidemic surveillance series. The Poisson models were severely overdispersed and produced unrealistically narrow confidence intervals. Negative binomial models substantially improved fit, and HAC standard errors further addressed residual serial dependence. The global MPC provided no evidence of one consistent log-linear trend and was sensitive to the treatment of the two initial zero-case months. The exploratory GAM also illustrated possible non-linearity (Figure 3), but its unfavorable k-check means that its detailed smooth shape cannot be treated as a validated representation of the underlying temporal process.

Poisson and negative binomial specifications are probability model families, whereas the analyses used a log link. The primary negative binomial models accommodated extra-Poisson variation, and HAC inference reduced the influence of short-range residual autocorrelation. These choices yielded more appropriately cautious uncertainty estimates; they do not eliminate all limitations of a short, aggregated time series or make the rate ratios unbiased causal effects. The GAM was retained solely as an exploratory visualization because the k = 10 basis-dimension diagnostic indicated potential underfitting.

Several limitations should be considered. Aggregated surveillance data are vulnerable to underreporting, reporting delays, revisions, changing testing criteria, and differences in access to diagnosis across pandemic phases. The ecological design precludes individual-level causal inference and adjustment for age, comorbidities, socioeconomic position, vaccination status, previous infection, clinical management, or infecting variant. Residual temporal autocorrelation remained detectable even after accounting for overdispersion; HAC inference reduces, but may not fully remove, its effect in a 48-month series. Period-specific CFR paired deaths and confirmed cases recorded in the same month rather than following a defined cohort, so delays from confirmation to death can distort short-term monthly CFR changes.

Two zero-case months required a continuity correction, although exclusion of those months produced nearly identical CFR ratios. The global MPC was sensitive to treatment of the initial zero months. In addition, the GAM basis-dimension diagnostic suggested that k = 10 was insufficient; the GAM is therefore descriptive only and its detailed curve should not be interpreted inferentially. As emphasized in methodological analyses of observational studies, statistical sophistication does not overcome the structural limitations of the study design, and associations derived from non-randomized data require theoretically grounded and cautious interpretation [28]. Accordingly, the present findings should be understood as associational evidence of epidemiological divergence rather than proof of biological causation.

## 5. Conclusions

Between 2020 and 2023, Pernambuco experienced marked changes in reported COVID-19 incidence, mortality, and period-specific case fatality. Monthly negative binomial models with autocorrelation-consistent inference showed lower mortality and case fatality from 2022 onward, even though annual reported incidence peaked in 2022. The surveillance data therefore support an epidemiological decoupling between reported transmission and severe outcomes; they do not establish which biological, clinical, behavioral, or surveillance mechanisms produced this pattern.

Vaccination, accumulated immunity, circulating variants, improvements in clinical care, and evolving testing practices are plausible and potentially concurrent explanations that require direct evaluation using linked or covariate-enriched data. Continued subnational surveillance should integrate vaccination, variant, hospitalization, testing, and demographic information and use time-series methods that account jointly for overdispersion and temporal dependence.

## Figures and Tables

**Figure 1:**
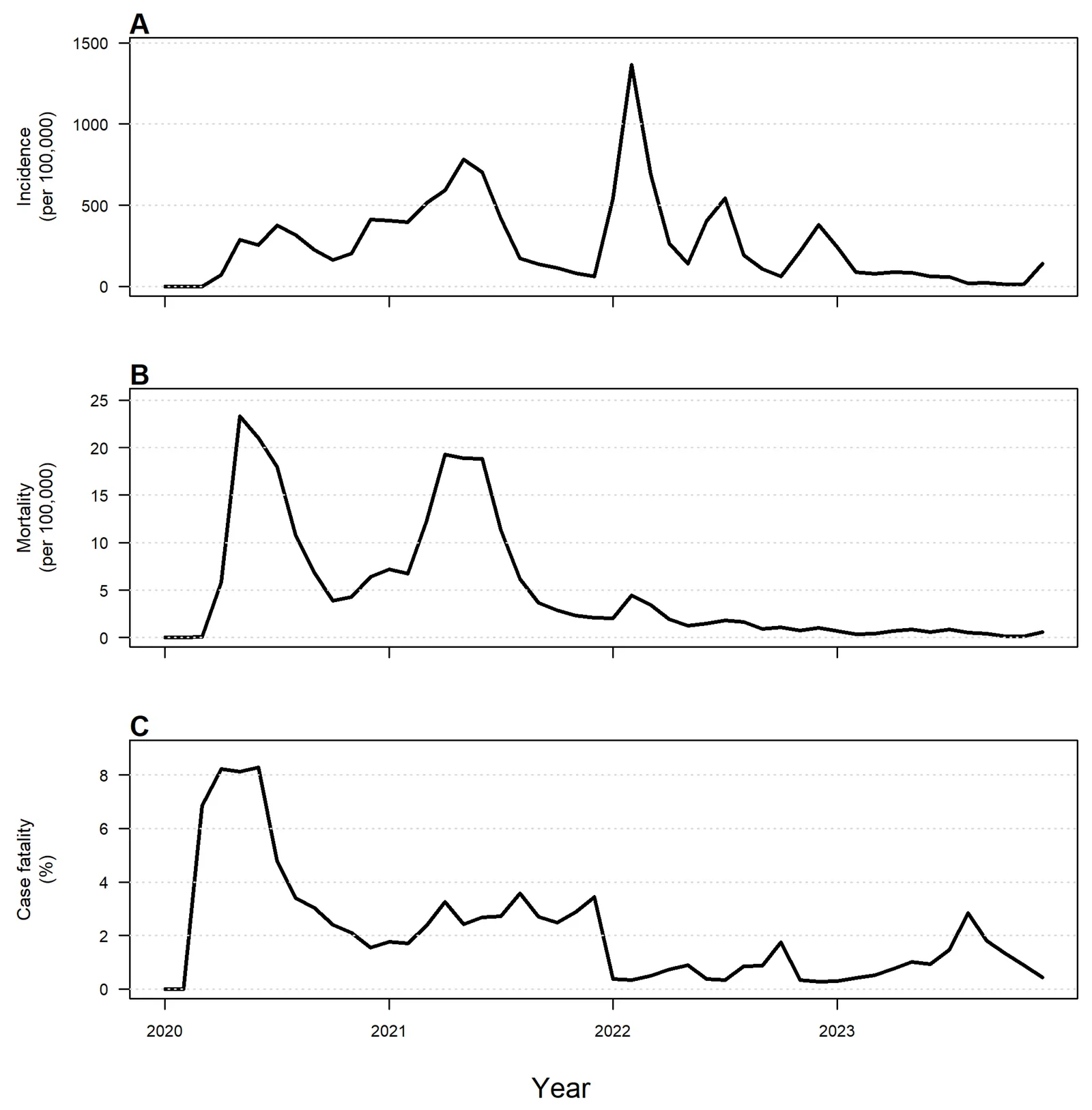
Monthly time series of COVID-19 epidemiological indicators in Pernambuco, Brazil (2020–2023). Note: (**A**) incidence rate per 100,000 inhabitants; (**B**) mortality rate per 100,000 inhabitants; and (**C**) case-fatality rate (%).

**Figure 2:**
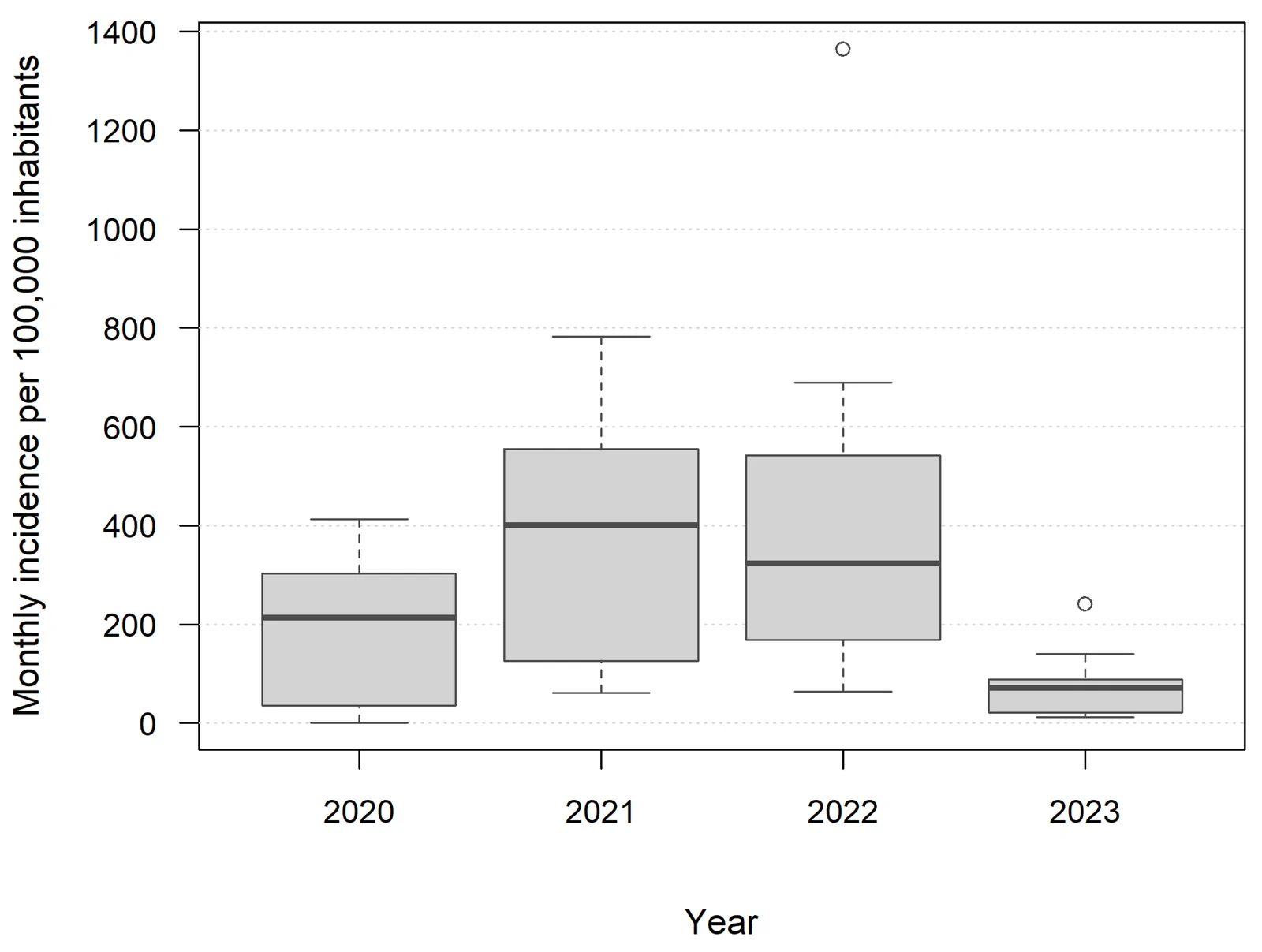
Annual distribution of monthly COVID-19 incidence rates in Pernambuco, Brazil (2020–2023). Boxplots summarize the distribution of monthly incidence rates within each calendar year. The horizontal line within each box represents the median, the box represents the interquartile range, and points beyond the whiskers indicate potential outliers.

**Figure 3:**
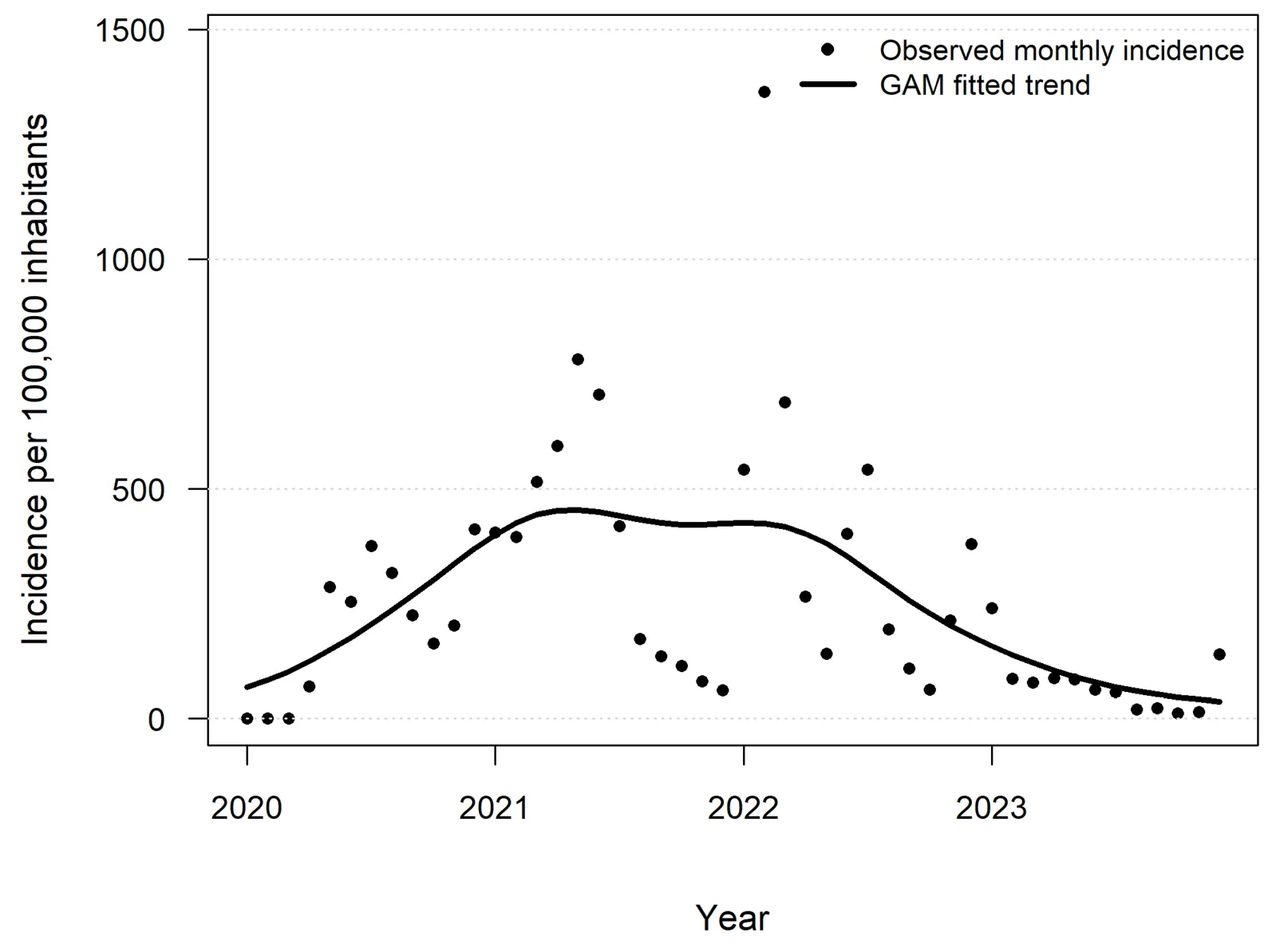
Exploratory non-linear temporal pattern of COVID-19 incidence in Pernambuco estimated using a generalized additive model (GAM).

**Table 1:** Confirmed cases, deaths, and annual cumulative epidemiological rates (Incidence, Mortality, and Case Fatality) for COVID-19 in Pernambuco, Brazil (2020–2023).

Year	Confirmed Cases	Confirmed Deaths	Incidence Rate (per 100k)	Mortality Rate (per 100k)	Case Fatality Rate (%)
2020	222,166	9654	2310.85	100.42	4.35
2021	423,515	10,793	4379.04	111.60	2.55
2022	477,254	2132	4905.65	21.91	0.45
2023	88,872	613	908.18	6.26	0.69

Incidence increased from 2310.85 per 100,000 in 2020 to 4379.04 per 100,000 in 2021 and peaked at 4905.65 per 100,000 in 2022. It then decreased to 908.18 per 100,000 in 2023. Mortality peaked in 2021 (111.60 deaths per 100,000), declined to 21.91 per 100,000 in 2022, and reached 6.26 per 100,000 in 2023. The period-specific CFR declined from 4.35% in 2020 to 2.55% in 2021 and 0.45% in 2022, followed by a modest increase to 0.69% in 2023; it therefore did not decline continuously across all four annual periods.

**Table 2:** Monthly descriptive statistics for COVID-19 epidemiological indicators in Pernambuco (2020–2023).

Indicator	Median	Interquartile Range (IQR)	Statistical Distribution
Incidence Rate (per 100k)	183.73	76.55–397.26	Descriptive measure; no probability distribution fitted
Mortality Rate (per 100k)	1.98	0.68–6.48	Descriptive measure; no probability distribution fitted
Case Fatality Rate (%)	1.62	0.52–2.75	Descriptive measure; no probability distribution fitted

**Table 3:** Primary negative binomial generalized linear models with HAC standard errors for monthly COVID-19 counts in Pernambuco, by calendar year (2020 as the reference).

Indicator	Model	Comparison	RR	95% CI	*p*-Value	Interpretation
Incidence Rate	NB-HAC	2021 vs. 2020	1.90	0.82–4.38	0.1327	No evidence of a difference
		2022 vs. 2020	2.13	0.93–4.86	0.0736	No evidence of a difference
		2023 vs. 2020	0.39	0.17–0.89	0.0247	Lower reported incidence
Mortality Rate	NB-HAC	2021 vs. 2020	1.11	0.43–2.89	0.8270	No evidence of a difference
		2022 vs. 2020	0.22	0.09–0.52	0.0006	Lower mortality
		2023 vs. 2020	0.06	0.03–0.14	<0.0001	Lower mortality
Period-specific CFR	NB-HAC	2021 vs. 2020	0.56	0.32–0.96	0.0363	Lower period-specific CFR
		2022 vs. 2020	0.13	0.07–0.25	<0.0001	Lower period-specific CFR
		2023 vs. 2020	0.21	0.10–0.47	0.0001	Lower period-specific CFR

**Table 4:** Temporal trend of monthly COVID-19 incidence estimated by log-linear regression in Pernambuco (2020–2023).

Indicator	Monthly Percent Change (MPC, %)	Moving-Block Bootstrap 95% CI	*p*-Value	Interpretation
Reported incidence	+2.52	−5.42 to +11.00	0.5152	No evidence of a consistent global log-linear trend; trajectory is oscillatory

## Data Availability

Publicly available datasets were analyzed in this study. Data can be accessed via official Ministry of Health platforms (https://covid.saude.gov.br/ accessed on 23 February 2026).

## Supplementary Materials

The following supporting information can be downloaded at: https://www.mdpi.com/article/10.3390/epidemiologia7050134/s1, Table S1, Comparative sensitivity analysis of Poisson and negative binomial models for COVID-19 incidence, mortality, and case fatality in Pernambuco, Brazil, 2020–2023; Table S2, Comparison of model fit and information criteria across Poisson, quasi-Poisson, and negative binomial specifications.
