# Supplementary material for: Epidemiological Decoupling of COVID-19 in Pernambuco, Brazil (2020–2023): A Subnational Time-Series Risk Analysis Using Generalized Models
Source: Epidemiologia (Basel). 2026 Sep 26;7(5):134. doi: 10.3390/epidemiologia7050134 (PMC13703668; doi:10.3390/epidemiologia7050134)
Supplement: Supplementary file 1 [file epidemiologia-07-00134-s001.zip › epidemiologia-4536869-supplementary.pdf]

Supplementary Materials

Table S1- Comparative sensitivity analysis of Poisson and negative binomial models for COVID-19 incidence, mortality, and case fatality in Pernambuco, Brazil, 2020–2023.

Indicator	Model	Comparison	RR	CI95	p_value
Incidence rate	Poisson	2021 vs 2020	1.89	1.89 – 1.9	< 0.0001
Incidence rate	Poisson	2022 vs 2020	2.12	2.11 – 2.13	< 0.0001
Incidence rate	Poisson	2023 vs 2020	0.39	0.39 – 0.4	< 0.0001
Incidence rate	Negative binomial	2021 vs 2020	1.90	0.76 – 4.73	0.1690
Incidence rate	Negative binomial	2022 vs 2020	2.13	0.85 – 5.3	0.1054
Incidence rate	Negative binomial	2023 vs 2020	0.39	0.16 – 0.98	0.0454
Mortality rate	Poisson	2021 vs 2020	1.11	1.08 – 1.14	< 0.0001
Mortality rate	Poisson	2022 vs 2020	0.22	0.21 – 0.23	< 0.0001
Mortality rate	Poisson	2023 vs 2020	0.06	0.06 – 0.07	< 0.0001
Mortality rate	Negative binomial	2021 vs 2020	1.11	0.52 – 2.39	0.7854
Mortality rate	Negative binomial	2022 vs 2020	0.22	0.1 – 0.47	0.0001
Mortality rate	Negative binomial	2023 vs 2020	0.06	0.03 – 0.13	< 0.0001
Case-fatality rate	Poisson	2021 vs 2020	0.59	0.57 – 0.6	< 0.0001
Case-fatality rate	Poisson	2022 vs 2020	0.10	0.1 – 0.11	< 0.0001
Case-fatality rate	Poisson	2023 vs 2020	0.16	0.15 – 0.17	< 0.0001
Case-fatality rate	Negative binomial	2021 vs 2020	0.56	0.36 – 0.86	0.0074
Case-fatality rate	Negative binomial	2022 vs 2020	0.13	0.09 – 0.2	< 0.0001
Case-fatality rate	Negative binomial	2023 vs 2020	0.21	0.14 – 0.33	< 0.0001

Note: Sensitivity analyses were conducted by fitting both Poisson and negative binomial models to each indicator. Incidence models used monthly case counts as the outcome and log(population) as the offset. Mortality models used monthly death counts as the outcome and log(population) as the offset. Case-fatality models used monthly death counts as the outcome and log(cases + 0.5) as the offset. All models used a logarithmic link function. The comparison evaluates the robustness of the estimated rate ratios to alternative assumptions regarding the conditional variance of the count outcome. The Negative Binomial estimates presented here serve as the primary inferential models, incorporating robust standard errors to account for residual serial dependence as discussed in the main text.

Table S2. Comparison of model fit and information criteria across Poisson, quasi-Poisson, and negative binomial specifications.

Indicator	Distribution	AIC	BIC	Dispersion	AIC_BIC_status
Incidence rate	Poisson	702393.53	702401.02	15951.332	Defined
Incidence rate	Quasi-Poisson	NA	NA	15871.541	Not defined for quasi-likelihood
Incidence rate	Negative binomial	1058.99	1068.35	NA	Defined
Mortality rate	Poisson	15530.57	15538.05	345.248	Defined
Mortality rate	Quasi-Poisson	NA	NA	327.735	Not defined for quasi-likelihood
Mortality rate	Negative binomial	648.69	658.04	NA	Defined
Case-fatality rate	Poisson	4620.94	4628.43	97.302	Defined
Case-fatality rate	Quasi-Poisson	NA	NA	103.677	Not defined for quasi-likelihood
Case-fatality rate	Negative binomial	577.16	586.52	NA	Defined

Note: AIC = Akaike Information Criterion; BIC = Bayesian Information Criterion. Lower AIC and BIC values indicate comparatively better fit among models estimated for the same outcome using a full likelihood. AIC and BIC are reported for the Poisson and negative binomial models. They are not defined for the quasi-Poisson model because quasi-likelihood estimation does not specify a complete probability likelihood; therefore, these cells are reported as NA rather than assigning artificial information-criterion values. For quasi-Poisson models, the estimated dispersion parameter is provided as the relevant variance-inflation diagnostic. Incidence models used cases with log(population) as offset; mortality models used deaths with log(population) as offset; case-fatality models used deaths with log(cases + 0.5) as offset.
